# Case Report: early regression and bladder-intact survival after limited-course immunotherapy-based systemic therapy in two patients with clinically staged bulky muscle-invasive bladder cancer

**DOI:** 10.3389/fimmu.2026.1866469

**Published:** 2026-06-08

**Authors:** Su Zhang, Hanghao Zhang, Xingwang Dong, Zhen Qi, Yingjun Ma, Hong Chang, Ye Li, Wei Zhang

**Affiliations:** 1 Department of Urology, Lanzhou University Second Hospital, Lanzhou, China; 2 Cuiying Honors College, Lanzhou University, Lanzhou, China; 3 Department of Urology, Gannan Tibetan Autonomous Prefecture People’s Hospital, Hezuo, China

**Keywords:** bladder preservation, case report, early response, immunotherapy-based systemic therapy, muscle-invasive bladder cancer

## Abstract

**Background:**

Radical cystectomy remains a standard curative treatment for muscle-invasive bladder cancer (MIBC). Bladder preservation after systemic therapy without immediate surgery remains investigational and requires careful patient selection, rigorous reassessment, and structured surveillance. We report two cases of early tumor regression after limited-course immunotherapy-based systemic therapy, with bladder-intact survival during available follow-up.

**Case presentation:**

Two male patients aged 75 and 73 years with clinically staged bulky bladder tumors suspicious for MIBC (one cT3N0M0 and the other at least cT2N0M0) received limited-course PD-1 inhibitor-based systemic therapy without immediate radical cystectomy. Both patients showed marked regression after two treatment cycles: Case 1 received tislelizumab plus platinum-based chemotherapy, and Case 2 received toripalimab plus disitamab vedotin. In Case 2, post-treatment transurethral reassessment and multi-site biopsies showed no definite residual malignancy; one additional cycle was then administered. Radical cystectomy was recommended but declined in both cases. At approximately three years after treatment initiation, no clinically detectable recurrence was identified based on available, non-protocolized assessments, although interval cystoscopic surveillance was incomplete.

**Conclusion:**

These cases document unusually early regression after limited-course immunotherapy-based systemic therapy in clinically staged bulky bladder tumors. The findings remain hypothesis-generating and do not justify routine omission of radical cystectomy or abbreviated systemic therapy. Instead, they suggest that early response kinetics may identify exceptional responders who warrant rigorous multimodal reassessment, molecular characterization, and structured surveillance within future response-adapted bladder-preserving strategies.

## 1 Introduction

Muscle-invasive bladder cancer (MIBC) is associated with a substantial risk of progression and cancer-related death. Radical cystectomy with perioperative cisplatin-based therapy remains a standard curative approach for eligible patients (1). In selected patients, bladder-preserving trimodality therapy, consisting of maximal transurethral resection followed by chemoradiotherapy, is an established alternative; however, this strategy requires careful patient selection, planned local treatment, and structured surveillance (2).

The rapid evolution of immunotherapy-based combinations has led to increasingly frequent observations of deep tumor regression in the neoadjuvant setting (3). Nevertheless, most pivotal trials still integrate systemic therapy with planned radical cystectomy as the definitive local treatment, and omitting surgery based on systemic response alone remains investigational (4). We report two real-world cases of clinically staged bulky MIBC in which marked early regression occurred after only two cycles of immunotherapy-based systemic therapy, followed by bladder-intact survival during approximately three years of available follow-up. These cases are not presented to support routine omission of radical cystectomy, but rather to highlight early response kinetics as a potential signal for rigorous multimodal reassessment, molecular characterization, and structured surveillance when management deviates from established treatment pathways.

## 2 Case presentation

### 2.1 Case 1

**Figure 1:**
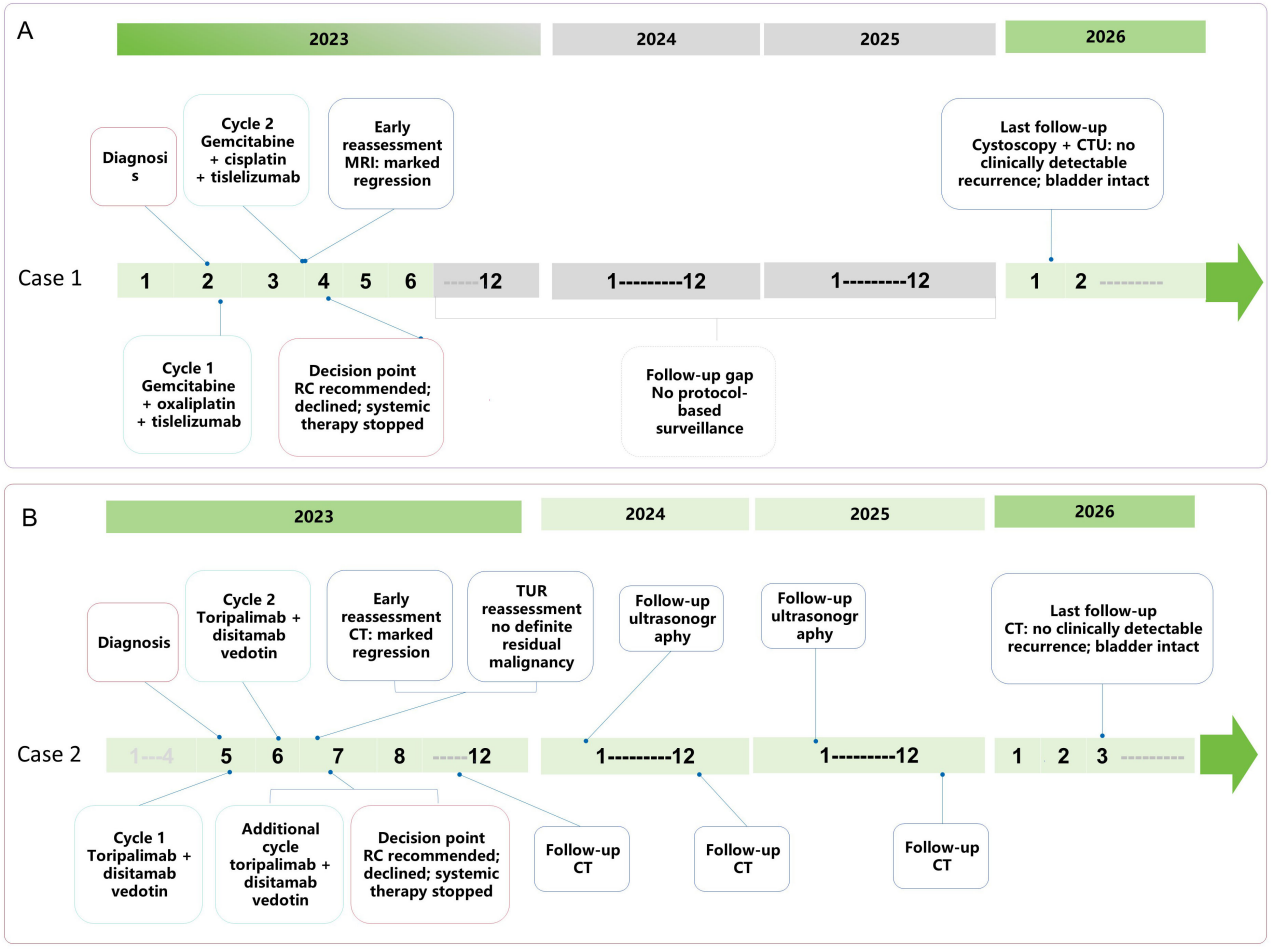
Clinical timelines of diagnosis, treatment, response assessment, and follow-up in the two cases. **(A)** Case 1 received two cycles of tislelizumab-based systemic therapy, followed by marked MRI regression. Radical cystectomy was recommended but declined, and no protocol-based surveillance was performed. Last follow-up in January 2026 showed no clinically detectable recurrence with bladder preservation. **(B)** Case 2 received two cycles of toripalimab plus disitamab vedotin, followed by marked CT regression and negative sampled TUR reassessment. One additional cycle was administered. Last follow-up in March 2026 showed no clinically detectable recurrence with bladder preservation.

A 75-year-old man presented in February 2023 with a 6-month history of intermittent painless gross hematuria. His medical history included a 3-year history of hypertension with suboptimal blood pressure control. He had no smoking history or known medication allergies. His Eastern Cooperative Oncology Group (ECOG) performance status was 1. Physical examination was unremarkable, with no abdominal tenderness, palpable abdominal or suprapubic mass, superficial lymphadenopathy, or lower-extremity edema. Laboratory testing showed mildly impaired renal function, with a serum creatinine level of 134 μmol/L. The clinical timeline for this patient is shown in **Figure 1A**.

**Figure 2:**
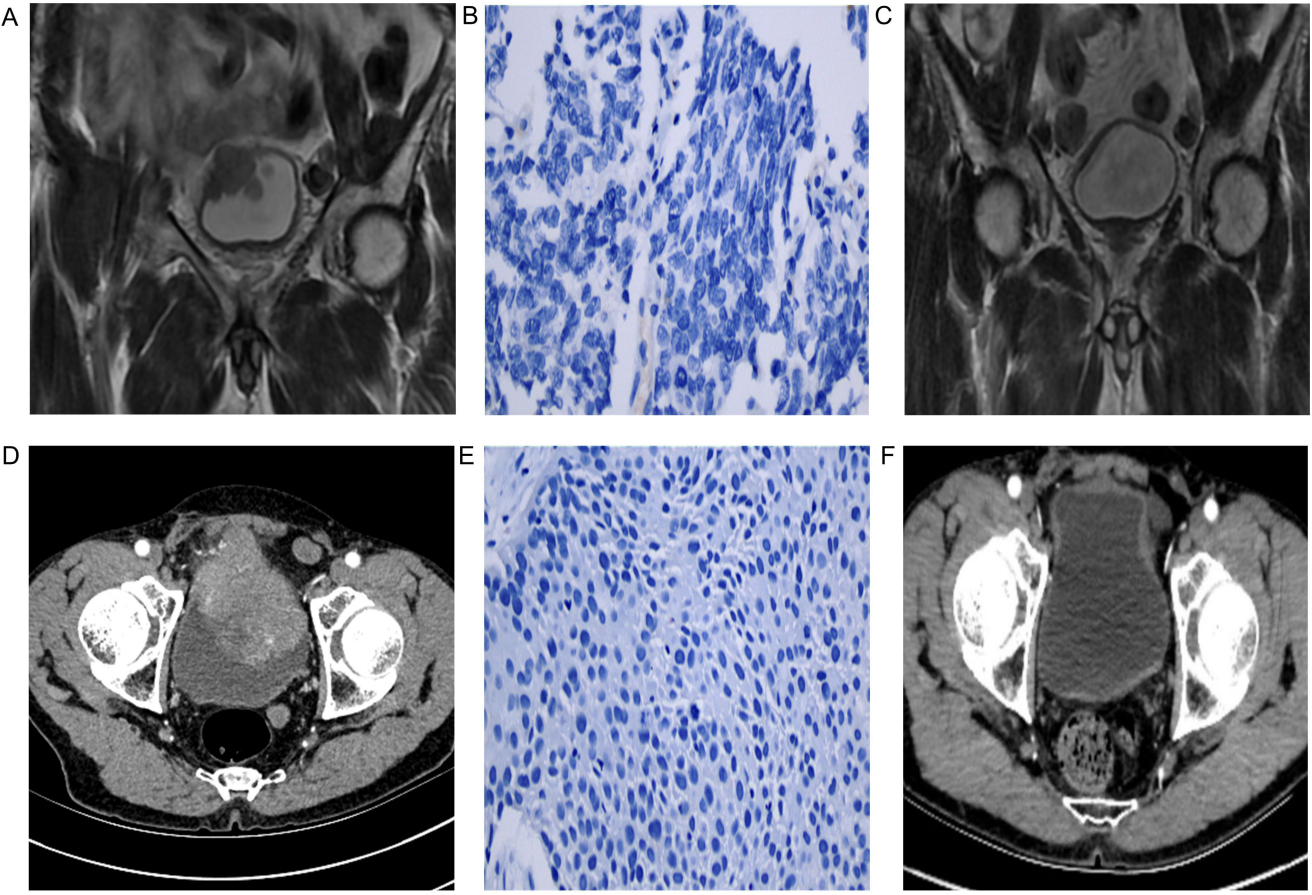
Baseline and post-treatment radiologic findings and immunohistochemical features of the two cases. **(A–C)** Case 1: **(A)** Baseline pelvic MRI showing a bulky bladder mass. **(B)** PD-L1 immunohistochemical staining showing CPS <1 (×200). **(C)** Post-treatment pelvic MRI obtained after two cycles of tislelizumab-based systemic therapy, approximately 57 days after treatment initiation, showing marked radiologic regression. **(D–F)** Case 2: **(D)** Baseline contrast-enhanced CT showing a large broad-based bladder tumor. **(E)** HER2 immunohistochemical staining showing negative expression with an IHC score of 0 (×200). **(F)** Post-treatment CT obtained after two cycles of toripalimab plus disitamab vedotin, approximately 60 days after treatment initiation, showing marked interval regression.

Initial CT urography demonstrated a bulky lobulated bladder mass measuring approximately 70 × 38 × 64 mm, with indistinct planes along the right and posterior bladder wall and involvement of the distal right ureter, accompanied by mild right hydroureteronephrosis. Pelvic MRI showed marked thickening of the right and posterior bladder wall involving the mucosal and muscular layers, with probable extravesical extension; the lesion was assigned a VI-RADS score of 5 (**Figure 2A**). No radiologically enlarged pelvic lymph nodes or distant metastases were identified on chest or abdominal imaging. Cystoscopy revealed multiple elevated intravesical lesions that were not amenable to maximal transurethral resection of bladder tumor (TURBT); diagnostic transurethral resection and biopsy were therefore performed. Histopathological examination confirmed high-grade urothelial carcinoma. Immunohistochemistry showed a PD-L1 combined positive score (CPS) <1 (**Figure 2B**) and a Ki-67 labeling index of 30%. Although muscularis propria was not identified in the biopsy specimen, the combined imaging findings, together with the absence of radiologically enlarged pelvic lymph nodes or distant metastases, supported a clinical stage of cT3N0M0.

Given the clinically advanced local disease, impaired renal function at presentation, and the patient’s persistent reluctance to undergo definitive local treatment, management was discussed in a multidisciplinary setting. Radical cystectomy was considered during initial treatment planning, but the patient remained hesitant to proceed with surgery. Bladder-preserving radiotherapy was also discussed but was not acceptable to the patient. An individualized platinum-based chemoimmunotherapy regimen was initiated. Systemic therapy was started on February 9, 2023. Because renal function was impaired at treatment initiation, the first cycle consisted of gemcitabine (1.2 g on day 1), oxaliplatin (150 mg on day 2), and tislelizumab (200 mg on day 7). After renal function improved, with serum creatinine decreasing to approximately 70 μmol/L, the second cycle was started on March 31, 2023, and treatment was adjusted to gemcitabine plus cisplatin (gemcitabine 1.2 g on days 1 and 8; cisplatin 90 mg on day 2) combined with tislelizumab (200 mg). Treatment was generally tolerated without grade 3 or higher adverse events, although nausea and vomiting were observed.

Pelvic MRI performed on April 9, 2023, after completion of two cycles, showed marked radiologic regression (**Figure 2C**). The previously visible bladder mass was no longer identified, and only focal bladder wall thickening remained at the original tumor site. Radical cystectomy was revisited and again recommended in light of this response; however, the patient declined definitive surgery. He also refused further systemic therapy because of treatment-related nausea and vomiting. No repeat transurethral resection or protocol-based post-treatment biopsy was performed. Post-treatment response in this case was therefore documented radiologically rather than pathologically.

The patient subsequently received no further treatment. Regular protocol-based surveillance, including scheduled cystoscopy and interval imaging, was recommended but was not completed because the patient did not return for follow-up during the intervening period. In January 2026, approximately three years after treatment initiation, he underwent late reassessment. Cystoscopy showed no visible abnormality. CT urography demonstrated adequate bladder filling, with no intraluminal abnormal density or filling defect, and no bladder wall thickening, abnormal enhancement, or enlarged pelvic lymph nodes. The perivesical fat plane was preserved, and no abnormality was identified in the upper urinary tract. At the latest reassessment, he reported no urinary symptoms and remained asymptomatic. Continuous recurrence-free status during the intervening period could not be verified because surveillance was not protocolized; however, no clinically detectable recurrence was identified on available late reassessment, and bladder-intact survival was maintained.

### 2.2 Case 2

A 73-year-old man was initially diagnosed in May 2023 after presenting with a 5-month history of intermittent painless gross hematuria. His medical history was significant for chronic obstructive pulmonary disease and hypertension. His ECOG performance status was 1. Physical examination was unremarkable, with no abdominal tenderness, palpable abdominal or suprapubic mass, superficial lymphadenopathy, or lower-extremity edema. The clinical timeline for this patient is shown in **Figure 1B**.

Contrast-enhanced CT and ultrasonography demonstrated a large broad-based solid bladder tumor measuring approximately 93 × 48 × 56 mm, predominantly involving the anterior wall and bladder dome (**Figure 2D**). Imaging showed focal disruption of the normal bladder wall architecture at the tumor base and mild strand-like blurring of the adjacent perivesical fat, raising strong concern for deep local invasion with possible focal extravesical extension. No radiologically enlarged pelvic lymph nodes or distant metastases were identified on chest or abdominal imaging. Cystoscopic examination confirmed a large solid tumor that was not amenable to maximal transurethral resection because of its size, broad base, and anatomical extent; biopsy was therefore performed for diagnostic purposes. Histopathological examination confirmed high-grade urothelial carcinoma. Immunohistochemical assessment showed HER2-negative status (IHC score 0, **Figure 2E**), PD-L1 CPS <1, and a Ki-67 labeling index of approximately 60%. Repeat HER2 immunohistochemical staining performed on the initial diagnostic biopsy specimen confirmed HER2 negativity, with an IHC score of 0. Although muscularis propria was not identified in the biopsy specimen, the lesion was clinically staged as at least cT2N0M0 on the basis of its large broad-based configuration, focal loss of normal bladder wall architecture, and imaging concern for perivesical extension.

Given the patient’s comorbidities, the technical infeasibility of maximal transurethral resection, and his persistent reluctance to undergo definitive local treatment, management was discussed in a multidisciplinary setting. Radical cystectomy was considered during initial treatment planning, but the patient remained hesitant to proceed with surgery. Bladder-preserving radiotherapy was also discussed but was not acceptable to the patient. He also declined chemotherapy. An individualized systemic treatment strategy was adopted. The first cycle of toripalimab (160 mg) combined with disitamab vedotin (120 mg) was administered on May 27, 2023, and the second cycle was administered on June 27, 2023. Treatment was well tolerated, and no grade 3 or higher adverse events were observed.

Repeat CT on July 27, 2023, showed marked interval regression of the bladder lesion, with only slight focal thickening remaining at the original tumor site (**Figure 2F**). He subsequently underwent transurethral reassessment with resection of the original tumor bed and multi-site biopsies for response evaluation. Pathological examination showed bladder tissue with multifocal necrosis and abundant foam cell infiltration, without definite evidence of residual malignancy in the sampled specimens, consistent with post-treatment changes. Radical cystectomy was revisited after response reassessment and was again recommended; however, the patient declined definitive surgery. One additional cycle of toripalimab plus disitamab vedotin was then administered, after which he refused further systemic treatment and did not undergo radical cystectomy.

Follow-up was conducted in routine clinical practice and consisted mainly of annual CT and ultrasonography rather than protocol-based cystoscopic surveillance. Urinalysis remained unremarkable, and the patient reported no urinary symptoms. At the last follow-up in March 2026, approximately three years after treatment initiation, repeat imaging showed no clinically detectable recurrence. He reported no urinary symptoms, remained in good general condition, and expressed willingness to continue clinical surveillance. Although long-term cystoscopic surveillance was unavailable, post-treatment transurethral reassessment and serial imaging supported bladder-intact survival during the available follow-up.

## 3 Discussion and conclusions

The most notable feature of the present report is that both patients with clinically staged bulky urothelial carcinoma of the bladder showed marked early regression after only two cycles of immunotherapy-based systemic therapy and subsequently maintained bladder-intact survival during the available follow-up. This pattern was observed with two distinct regimens: tislelizumab combined with platinum-based chemotherapy (Case 1) and toripalimab combined with disitamab vedotin (Case 2). Despite a substantial baseline local tumor burden, both patients exhibited an exceptionally rapid response within a short treatment interval, suggesting that early regression kinetics may reflect marked individual treatment sensitivity not fully captured by current treatment frameworks.

These cases also differed from both trimodality therapy and emerging post-neoadjuvant bladder-sparing pathways, both of which rely on patient selection rather than indiscriminate bladder preservation. Current European Association of Urology guidelines and major comparative studies, such as the series by Zlotta et al., typically restrict trimodality therapy to patients with favorable local characteristics, such as solitary, unifocal cT2-T3a tumors (<7 cm), absence of extensive carcinoma *in situ*, adequate bladder function, and no hydronephrosis (1, 2). Similarly, emerging post-neoadjuvant bladder-sparing pathways have generally relied on risk-adapted selection rather than response alone. In the RETAIN-1 trial, active surveillance was considered only for patients with predefined DNA damage repair gene alterations and cT0 status after neoadjuvant chemotherapy, underscoring the role of biomarker-informed and post-treatment clinical selection in cystectomy-sparing strategies (5). In contrast, our cases presented with adverse baseline features: both tumors were markedly bulky, unsuitable for debulking TURBT, and showed imaging evidence of possible extravesical extension or associated upper tract dilatation. These differences do not challenge the importance of patient selection, but they suggest that adverse baseline local features may not fully capture the potential for early deep response to contemporary systemic therapy. This distinction may be relevant when considering how baseline anatomical criteria and post-treatment response should be weighted in future bladder-preserving strategies.

An additional point of context is treatment intensity and subsequent local management. In pivotal neoadjuvant and perioperative studies, systemic therapy is generally delivered for at least three to four cycles and is embedded within planned local treatment pathways, most commonly radical cystectomy. The KEYNOTE-905/EV-303 perioperative EV/pembrolizumab study provides important context for the expanding role of ADC-immunotherapy combinations in muscle-invasive bladder cancer, evaluating this regimen within a planned radical cystectomy-based treatment pathway. In that study, patients in the EV/pembrolizumab arm received three neoadjuvant cycles before radical cystectomy and pelvic lymph node dissection, followed postoperatively by up to 14 adjuvant cycles of pembrolizumab, including six cycles in combination with EV (6). Studies that more directly explored cystectomy deferral or bladder preservation have also generally incorporated both a longer induction phase and additional post-response treatment before or during surveillance. For example, in HCRN GU16-257, patients received four cycles of gemcitabine-cisplatin plus nivolumab before formal clinical complete response assessment; those who achieved a stringent clinical complete response and opted for bladder preservation then received eight additional doses of nivolumab administered every two weeks, together with protocol-based surveillance (7). In the present report, this early regression occurred after only two cycles; Case 1 received no further treatment, and Case 2 received only one additional cycle after negative pathological reassessment of sampled tumor-bed specimens, without maximal debulking TURBT, definitive radiotherapy, or radical cystectomy in either case. In the absence of definitive complete-response confirmation, abbreviated systemic therapy without local consolidation could risk undertreating occult residual disease. Taken together, these observations should not be interpreted as evidence that abbreviated therapy is sufficient, but rather as a rationale for evaluating early response kinetics as a trigger for rigorous multimodal restaging before any deviation from standard local treatment is considered.

Beyond differences in selection and treatment intensity, the biomarker findings require cautious interpretation. Both tumors had PD-L1 CPS <1, although early neoadjuvant data from PURE-01 suggested higher pathological response rates among patients with higher CPS values (8), whereas later studies, including ABACUS, did not establish PD-L1 as a consistent standalone predictor of long-term benefit (9). The HER2 finding in Case 2 also requires caution. Most neoadjuvant studies of disitamab vedotin in localized MIBC have focused on HER2-expressing disease, and a recent multicenter real-world series associated HER2 overexpression with a higher likelihood of response (10); however, activity of disitamab vedotin has also been reported in locally advanced or metastatic urothelial carcinoma with HER2-low or HER2-null expression, including HER2 IHC 0 disease (11). Therefore, the marked regression observed in the HER2-negative case should be viewed as an outlying clinical observation, not as evidence that HER2 status is irrelevant in localized disease. Taken together, baseline PD-L1 CPS and HER2 status alone do not fully explain the early responses observed in these patients. Future studies of such exceptional responders should integrate baseline molecular profiling, such as DNA damage repair alterations, tumor mutational burden, molecular subtype, and immune-response signatures, with longitudinal molecular monitoring using circulating tumor DNA or urine-based tumor DNA. This is particularly relevant given the emerging role of ctDNA-guided treatment in MIBC, as illustrated by IMvigor011 (12). In the present report, these analyses were not performed, limiting our ability to biologically explain the observed responses and underscoring the need for molecular biomarkers to support rigorous multimodal reassessment and structured bladder-preserving surveillance.

Several limitations substantially restrict the interpretation of these cases. This report includes only two patients, both of whom declined radical cystectomy and were managed outside a protocol-defined bladder-preserving pathway, limiting generalizability and introducing selection bias. Both tumors were clinically staged as muscle-invasive or locally advanced disease on the basis of imaging and cystoscopic findings, because muscularis propria was not identified in the initial diagnostic biopsy material. Post-treatment response assessment was less complete in Case 1, in which regression was documented radiologically without repeat transurethral reassessment or biopsy; in contrast, negative sampled reassessment of the tumor bed in Case 2 provided pathological support for local response. Follow-up was also non-protocolized, with a prolonged interval without surveillance in Case 1 and mainly imaging-based follow-up in Case 2; therefore, although no clinically detectable recurrence was identified on available assessments, occult intravesical recurrence during surveillance gaps cannot be fully excluded. Despite these limitations, these cases remain informative as real-world examples of exceptional early response and highlight the need for systematic molecular characterization, rigorous reassessment, and structured surveillance in future response-adapted bladder-preserving studies.

In conclusion, these two cases suggest that unusually early and deep regression may occur after limited-course immunotherapy-based systemic therapy in clinically staged bulky bladder tumors, even in the absence of favorable baseline local features. However, given the non-standard treatment courses, incomplete pathological confirmation in Case 1, and non-protocolized surveillance, these findings remain hypothesis-generating and should not be used to support routine omission of radical cystectomy or abbreviated systemic therapy. The main scientific implication is that early response kinetics, if validated together with molecular and longitudinal biomarkers, may contribute to more precise response-adapted bladder-preserving strategies, in which treatment intensity and surveillance are individualized to balance oncologic safety, treatment burden, and the possibility of bladder preservation.

## Patient perspective

Both patients expressed a preference to avoid radical cystectomy and preserve the bladder after repeated discussion of standard treatment options and potential oncologic risks. At the latest follow-up, both reported no urinary symptoms and were satisfied with their bladder-intact status. They were advised to continue structured clinical surveillance.

## Data Availability

The original contributions presented in the study are included in the article/supplementary material. Further inquiries can be directed to the corresponding author.
